# Determination of Ebastine in Pharmaceutical Formulations by HPLC

**DOI:** 10.4103/0250-474X.43022

**Published:** 2008

**Authors:** S. L. Prabu, C. Dinesh Kumar, A. Shirwaikar, Annie Shirwaikar

**Affiliations:** 1 Department of Pharmaceutical Quality Assurance, Manipal College of Pharmaceutical Sciences, Manipal-576 104, India; 2 Department of Pharmacognosy, Manipal College of Pharmaceutical Sciences, Manipal-576 104, India; Department of Pharmaceutics, Manipal College of Pharmaceutical Sciences, Manipal-576 104, India

**Keywords:** Ebastine, reverse-phase liquid chromatography, solid dosage form

## Abstract

A simple, precise and rapid RP-HPLC method was developed for the determination of ebastine in pharmaceutical formulations. The method was carried out on a Phenomenex RP-C18 column using a mixture of methanol and water (90:10) and detection was done at 262 nm. The linearity range was 5-100 μg/ml. The intra-day and inter-day precision were in the range of 0.22% to 0.49% and 0.24% to 0.73%, respectively.

Ebastine is a new generation of antihistamines which has potent and selective histamine H_1_-receptor antagonistic effect, but negligible anticholinergic and antiserotonergic properties^1^–^2^. Ebastine is effective for the treatment of chronic idiopathic urticaria and allergic diseases with once daily regimen, and the antihistaminic action is mainly induced by the active metabolite, carebastine that is rapidly generated in the small intestine and in the liver^3^.

To date, a couple of chromatographic methods have been reported to quantify ebastine and its metabolites in physiological sample^4^. Kang *et al* and Rohatagi *et al*, recently improved the assay methodology for ebastine and carebastine by using a tandem mass spectrometry in human plasma^4^,^5^. However, till date no assay procedure has been reported for the determination of this drug in pharmaceutical formulations. Hence, there is a need to develop a simple assay procedure for the determination of this drug in pharmaceutical formulations. The availability of an HPLC method with high sensitivity and selectivity would be very useful for the determination of ebastine in pharmaceutical dosage forms.

Ebastine (assigned purity 99.8%) was a gift sample from Eros Pharma, Bangalore, India. HPLC grade methanol and water were procured from Ranbaxy Fine Chemicals Limited, SAS Nagar, India and Qualigens Chemicals, India respectively. Commercially available ebastine tablets, claimed to contain 10 and 20 mg of ebastine, respectively, were procured from the local Pharmacy. Quantitative HPLC was performed on an isocratic high pressure liquid chromatograph (Shimadzu HPLC Class 10A Series) with two LC-10AT pumps, using a fixed wavelength guided by a programmable UV/Vis detector (SPD-10A). The column used was Phenomenex RP-C18 (250 mm × 4.6 mm i.d., Particle size 5 μ ). The HPLC system was equipped with the software, Class LC-10AT series, version 5.03 (Shimadzu).

For HPLC, the mobile phase, methanol:water (90:10), was filtered before use through a 0.45 μm membrane filter. It was degassed with a helium spurge for 15 min and pumped from the respective solvent reservoirs to the column at a flow rate of 1.5 ml/min. The run time was set at 15 min and the column temperature was maintained at 25°. The volume of injection loop was 20 μl. Prior to the injection of the drug solutions, the column was equilibrated for at least 45 min with the mobile phase flowing through the systems. The eluent was monitored at 262 nm and the data acquired was stored and analyzed with the software.

A stock solution of the drug, prepared by dissolving 50 mg of ebastine in a 50 ml volumetric flask containing methanol (HPLC grade), was sonicated for about 10 min and then finally the volume was made up using methanol. Daily working standard solutions of ebastine were prepared by suitable dilution of the stock solution with the mobile phase. Five sets of the drug solutions were prepared in the mobile phase containing ebastine at a concentration range of 5-100 μg/ml. Each of these drug solutions (20 μl) in duplicate was injected, into the column and the peak area and retention time was recorded and analyzed.

Not less than twenty tablets were weighed to obtain the average tablet weight. The tablets were then powdered. A sample of the powdered tablets, claimed to contain 25 mg of the active ingredient was taken in a clean and dry 50 ml volumetric flask. The contents of the flask were dissolved in a small quantity of methanol and the volume was made up with the same. This mixture was shaken well and then filtered through a 0.45 μm membrane filter. An appropriate dilution was made with the mobile phase to get a concentration of 100 μg/ml. Various volumes of this aliquot were diluted, to obtain concentrations ranging between 80-120% of test concentrations, with mobile phase. All determinations were conducted in triplicate. The same procedure was used to estimate the concentration of the drug in three different strengths of ebastine tablets.

The aim of our study was to develop a simple, precise and rapid RP-HPLC method for the quantitative analysis of ebastine in its pharmaceutical dosage forms. The retention time of ebastine was found to be 9.2-9.3 min. System performance parameters such as capacity factor, asymmetry and number of theoretical plates were found to be 3.79, 1.44 and 7590, respectively. The calibration curve of ebastine was constructed by plotting the area of ebastine against concentration. It was found to be linear with a correlation coefficient of 0.9996, the representative linear regression equation being Y = 24876X+21148. This method was also validated for its intra and inter-day precision studies, the relative standard, based on the peak area for six triplicate injections for intra-day was found to be between 0.22% and 0.49% and the inter-day assay precision (3 days, n-5) was expressed as relative standard deviation, the range being between 0.24% and 0.73%.

For the two different strengths of ebastine tablets used viz, 10 mg and 20 mg the results were found to be 9.91and 20.07 mg. The absence of interfering peaks in the chromatogram suggests that the tablet excipients do not interfere with the estimation of the drug by the proposed HPLC method. It was also observed that there was a substantial recovery of ebastine when a known amount of the drug solution was added to a powdered sample of the tablet dosage form. The average recovery was found to be 99.63%. The limit of detection and limit of quantification was found to be 50 ng/ml and 100 ng/ml, respectively.

The results of our study indicate that the proposed RP-HPLC method is simple, rapid, precise and accurate. The method could be of immense use for the determination of ebastine in its pharmaceutical dosage forms.
